# Clinicopathologic ımpact and prognostic value of lymphovascular space ınvasion within the MMRd molecular subgroup of endometrial carcinoma

**DOI:** 10.61622/rbgo/2026rbgo77

**Published:** 2026-10-30

**Authors:** Esra Keles, Mustafa Maraşlı, Meziha Taşyurek, Elif Unlugedik Sayın, Öncel İpekçi, Mervenur Şahin, Ugur Kemal Öztürk, Fatih Şanlıkan, Murat Api

**Affiliations:** 1 University of Health Sciences Kartal Dr. Lütfi Kırdar City Hospital Department of Gynecologic Oncology Istanbul Turkey Kartal Dr. Lütfi Kırdar City Hospital, Department of Gynecologic Oncology, University of Health Sciences, Istanbul, Turkey.; 2 University of Health Sciences Kartal Dr.Lütfi Kırdar City Hospital Department of Obstetrics and Gynecology Istanbul Turkey Kartal Dr.Lütfi Kırdar City Hospital, Department of Obstetrics and Gynecology, University of Health Sciences, Istanbul, Turkey; 3 University of Health Sciences Kartal Dr.Lütfi Kırdar City Hospital Department of Pathology Istanbul Turkey Kartal Dr.Lütfi Kırdar City Hospital, Department of Pathology, University of Health Sciences, Istanbul, Turkey

**Keywords:** Endometrial neoplasms, DNA mismatch repair, Mismatch repair endonuclease PMS2, Prognosis, Molecular classification

## Abstract

**Objective:**

To investigate the clinicopathological significance and prognostic impact of lymphovascular space invasion (LVSI) within the mismatch repair-deficient (MMRd) molecular subgroup of endometrial cancer.

**Methods:**

A retrospective study was conducted on patients with histologically confirmed endometrial carcinoma who underwent primary surgical staging between January 2017 and December 2020. Immunohistochemical analysis of mismatch repair proteins (MLH1, MSH2, MSH6, and PMS2) was performed to classify tumors into MMR-proficient or MMR-deficient (MMRd) subgroups. LVSI status was independently reviewed and classified as absent, focal, or substantial by two gynecologic pathologists. Clinicopathologic findings and follow-up data were extracted from the institutional electronic medical records.

**Results:**

A total of 214 patients with a mean age 60.61±9.70 years were included, with a median follow-up of 24.6 months (IQR: 24.7 months). Fifteen death events (7.0%) were recorded during the study period. LVSI was identified in 42 patients (19.6%), including 26 with focal and 16 with substantial involvement. LVSI-positive tumors were significantly associated with larger tumor size (p<0.001), high-grade histology (p<0.001), non-endometrioid subtype (p<0.001), lymph node metastasis (p<0.001), and advanced FIGO stage (p<0.001). Among the 70 patients (32.7%) with MMRd tumors, LVSI was observed in 14 cases (20.0%) and remained significantly associated with aggressive features, including larger tumor size (p <0.001), high-grade histology (p = 0.010), advanced stage (p = 0.011), and lymph node involvement (p <0.001). However, overall survival did not differ significantly between LVSI-positive and LVSI-negative MMRd patients (log-rank p = 0.213).

**Conclusion:**

LVSI is associated with adverse clinicopathologic features in endometrial cancer, including within the MMRd molecular subgroup. However, its presence does not appear to independently influence overall survival in MMRd patients.

## Introduction

Endometrial carcinoma (EC) represents the most prevalent gynecologic malignancy in developed nations, with rising incidence worldwide.^(1)^ Historically, prognostication and treatment have relied on established clinicopathologic parameters, including International Federation of Gynecology and Obstetrics (FIGO) stage, tumor grade, histologic subtype, depth of myometrial invasion, and lymphovascular space invasion (LVSI).^(2)^ However, the paradigm of EC classification has evolved with the incorporation of molecular profiling, which stratifies tumors into four prognostically distinct subtypes: POLE ultramutated (POLEmut), mismatch repair deficient (MMRd), p53 abnormal (p53abn), and no specific molecular profile (NSMP).^(3)^ This molecular framework, now embedded in the WHO 5th edition classification and international guidelines, including the 2023 FIGO staging revisions and 2023 ESGO/ESTRO/ESP recommendations, has enabled more individualized treatment strategies, particularly the use of immunotherapy in MMRd tumors.^(4)^

LVSI is a robust predictor of lymph node involvement, disease recurrence, and reduced survival in EC.^(5–7)^ Its clinical importance has been reaffirmed by the 2023 FIGO staging update, which now includes substantial LVSI as a risk-defining criterion. Nevertheless, emerging data suggest that the prognostic role of LVSI may be modulated by underlying molecular subtype, raising questions about its consistency across distinct molecular subgroups.^(8)^

MMRd, identified in approximately 15–30% of ECs, is characterized by microsatellite instability and often presents with low-grade, early-stage endometrioid histology.^(9)^ While MMRd is associated with favorable outcomes and predictive responsiveness to immune checkpoint inhibitors, its independent prognostic role remains debated, with some studies reporting neutral or even adverse impacts on long-term survival.^(10,11)^ This ambiguity underscores the need to better define how LVSI interacts with molecular alterations, particularly MMR status, to inform prognostication. Although prior molecular analyses have highlighted the prognostic relevance of LVSI across EC subtypes, studies specifically addressing its impact within each molecular class remain limited.

The present study aimed to (i) determine the prevalence of LVSI in relation to MMR status, (ii) characterize the clinicopathological correlates of LVSI, and (iii) assess the prognostic significance of LVSI within the MMRd molecular subgroup.

## Methods

### Study design and patient selection

This retrospective study comprised patients with a confirmed diagnosis of EC who underwent primary surgical staging surgery between January 2017 and December 2020 at the Gynecologic Oncology Department of a tertiary hospital. Inclusion criteria required histologically confirmed diagnosis of EC and complete MMR status data obtained through standardized immunohistochemical testing. Exclusion criteria included incomplete clinicopathological data or absence of MMR testing results. The study adhered to the Strengthening the Reporting of Observational Studies in Epidemiology (STROBE) guidelines for retrospective cohort investigations.

### Clinicopathological data collection

Comprehensive clinicopathological data were systematically extracted from the institutional electronic health records and pathology reports. Data elements included patient demographics (age at diagnosis, body mass index), tumor characteristics (size, grade, histologic subtype, FIGO stage, depth of myometrial invasion), treatment details (surgical approach, adjuvant therapy), and longitudinal follow-up information pertaining to patient survival outcomes.

Overall survival (OS) was selected as the primary endpoint because it represents a definitive, unambiguous outcome that can be reliably ascertained from institutional and national mortality registries, minimizing the risk of misclassification and recall bias. While recurrence-free survival (RFS) or progression-free survival (PFS) often provide earlier signals of treatment efficacy and biological aggressiveness, in retrospective single-center studies these endpoints can be inconsistently documented, particularly when patients receive follow-up care at external institutions or in community settings. OS was calculated from the date of primary staging surgery to the date of death from any cause. Patients alive at the end of the study period were censored at the date of last documented contact. The median follow-up time was 24.6 months (interquartile range: 24.7 months).

### Pathological Review and LVSI Assessment

All pathology slides pertaining to the included patients underwent meticulous review by two experienced gynecopathologists. Tissue samples were fixed in 10% neutral buffered formalin with standardized fixation times (6–48 hours) and processed according to College of American Pathologists (CAP) and European Society for Medical Oncology (ESMO) guidelines to ensure optimal tissue preservation and antigen integrity.

Tumor grade was classified according to the binary grading system recommended by FIGO, which categorizes endometrial carcinomas as low-grade (Grade 1 and Grade 2) or high-grade (Grade 3) based on architectural pattern and nuclear atypia.^(12)^ LVSI was further categorized based on its extent following standardized criteria: focal LVSI was defined as the presence of tumor cells within one to four lymphovascular spaces in the tissue sections examined, whereas substantial LVSI was characterized by the presence of tumor cells in five or more distinct lymphovascular spaces, or the presence of extensive intravascular tumor growth (Figure 1).^(12)^

**Figure 1:**
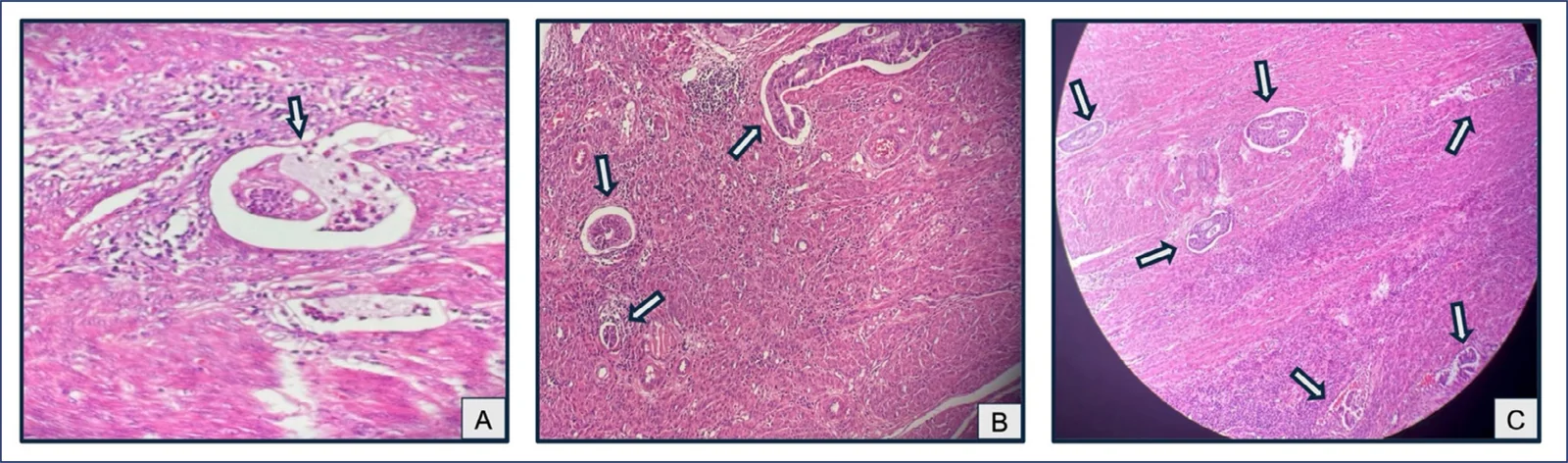
Histopathological assessment of lymphovascular space invasion (LVSI) in endometrial carcinoma.

(A) Representative image demonstrating tumor cells within an endothelial-lined vascular space, diagnostic of lymphovascular invasion (hematoxylin and eosin [H&E] stain, original magnification ×200). (B) Focal LVSI, defined as tumor involvement of fewer than 5 lymphovascular spaces, shown in an endometrioid endometrial carcinoma (H&E stain, original magnification ×100). (C) Substantial LVSI, characterized by tumor involvement of 5 or more lymphovascular spaces; this field demonstrates 6 involved vessels (H&E stain, original magnification ×40)

For the purposes of primary analysis, patients were classified as LVSI-positive if their tumor exhibited any evidence of LVSI (either focal or substantial), and LVSI-negative if no lymphovascular invasion was identified. Secondary analyses examined focal versus substantial LVSI as separate categories. Interobserver concordance for LVSI assessment was evaluated, and discordant cases were adjudicated through consensus review at a multiheaded microscope. The pathologists were blinded to clinical outcome data during the histopathological review process to minimize assessment bias.

### Molecular analysis

Mismatch repair status assessment was performed using standardized immunohistochemistry (IHC) protocols evaluating protein expression for four MMR proteins: MSH2, MSH6, MLH1, and PMS2. IHC staining was performed on an automated Ventana BenchMark ULTRA platform (Roche Diagnostics, Basel, Switzerland) using the following validated antibodies: MSH6 (clone SP93, rabbit monoclonal, ready-to-use, Ventana Medical Systems), PMS2 (clone A16-4, mouse monoclonal, ready-to-use, Ventana Medical Systems), MSH2 (clone G219-1129, mouse monoclonal, ready-to-use, Ventana Medical Systems), and MLH1 (clone M1, mouse monoclonal, ready-to-use, Ventana Medical Systems).

Standardized staining protocols included heat-induced epitope retrieval using Cell Conditioning 1 (CC1) solution (Ventana Medical Systems) and detection using the OptiView DAB IHC Detection Kit (Ventana Medical Systems). Internal positive controls for the immunostains were non-neoplastic endometrial epithelial cells, endometrial stromal cells, and tumor-infiltrating lymphocytes present within each tissue section. In the presence of appropriate positive internal controls, retained nuclear staining in tumor cells was interpreted as normal (intact) protein expression, indicating MMR proficiency. Complete absence of nuclear staining in tumor cells, with retained staining in internal controls, was interpreted as loss of protein expression, indicating MMR deficiency.

In accordance with WHO 5th edition classification guidelines,^(13)^ mismatch repair deficiency (MMRd) was defined as the loss of nuclear staining for at least one of the four MMR proteins (MLH1, MSH2, MSH6, or PMS2). Two independent pathologists reviewed the immunohistochemical staining results, with discrepancies resolved through consensus review. Equivocal or indeterminate staining patterns were subjected to repeat immunostaining or supplementary molecular testing when clinically indicated.

POLE exonuclease domain sequencing was not available in Turkey during the study period (2017–2020) and remains unavailable in routine clinical practice at present, which precluded assignment of cases to the POLE-ultramutated molecular subgroup. Consequently, molecular classification in this cohort was based on MMR status and p53 immunohistochemistry, allowing stratification into MMRd, p53-abnormal, and no specific molecular profile (NSMP) categories only.

### Statistical analysis

Statistical analysis was performed using IBM SPSS Statistics for Windows, Version 25.0. (IBM Corp., Armonk, NY, USA). Descriptive statistics were generated for all variables. Categorical variables were summarized as frequencies and percentages, and compared between groups using Pearson's chi-square test or Fisher's exact test. Continuous variables were assessed for normality of distribution using the Shapiro-Wilk test and visual inspection of histograms and Q-Q plots. Normally distributed continuous variables were reported as mean ± standard deviation (SD) and compared using independent samples t-tests or one-way analysis of variance (ANOVA) for multiple group comparisons. Non-normally distributed continuous variables (including tumor size, which exhibited significant right skewness) were reported as median with interquartile range (IQR: 25th–75th percentiles) and compared using the Mann-Whitney U test or Kruskal-Wallis test as appropriate.

Survival curves were generated using the Kaplan-Meier method, and between-group differences in survival distributions were assessed using the log-rank test. Multivariable Cox proportional hazards regression models were constructed to assess the independent prognostic effect of LVSI while adjusting for established confounding variables including FIGO stage (I/II vs. III/IV), tumor grade (low-grade vs. high-grade), histologic subtype (endometrioid vs. non-endometrioid), and MMR status (proficient vs. deficient). The proportional hazards assumption was evaluated using Schoenfeld residuals and log-minus-log survival plots. Hazard ratios (HR) with corresponding 95% confidence intervals (CI) and two-tailed *p*-values were reported for all Cox regression analyses. All statistical tests were two-sided, and statistical significance was defined as *p*<0.05.

### Ethical approval

Patients underwent surgical treatment between January 2017 and December 2020. The institutional review board (IRB) provided formal approval for the retrospective analysis of de-identified data on 26 February 2025 (IRB Protocol No. 2025/010.99/13/16) and granted a waiver of written informed consent for use of archived, anonymized medical records. The IRB approval date refers to authorization to perform the present retrospective analysis of historical data. All procedures were conducted in strict accordance with the ethical principles of the Declaration of Helsinki (2013 revision) and local regulatory requirements.

## Results

### Clinicopathological characteristics

A total of 214 patients with histologically confirmed EC met inclusion criteria and were included in the final analysis. Baseline patient and tumor characteristics are summarized in Table 1. The mean (±SD) age at diagnosis was 60.6 (±9.7) years (range: 32–85 years), and the mean body mass index (BMI) was 32.4 (±6.2) kg/m² (range: 18.5–54.2 kg/m²). The distribution of FIGO stage demonstrated that the majority of patients presented with early-stage disease: 75 patients (75.8%) were classified as FIGO stage I, 13 patients (13.1%) as stage II, 9 patients (9.1%) as stage III, and 2 patients (2.0%) as stage IV. Tumor grade distribution revealed that 94 patients (46.1%) had Grade 1 (G1) tumors, 85 patients (41.7%) had Grade 2 (G2) tumors, and 25 patients (12.3%) had Grade 3 (G3) high-grade tumors. Regarding histologic subtype, the majority of tumors were of the endometrioid subtype (n = 197, 92.1%), followed by carcinosarcoma (n = 7, 3.3%), serous carcinoma (n = 4, 1.9%), other subtypes (n = 5, 2.3%), and clear cell carcinoma (n = 1, 0.5%). The median tumor size was 12.0 mm (IQR: 8.0–22.0 mm), with a range from 2 mm to 180 mm. LVSI was identified in 42 patients (19.6% of the cohort). Among LVSI-positive cases, 26 patients (12.1% of total cohort) exhibited focal LVSI and 16 patients (7.5% of total cohort) exhibited substantial LVSI. Molecular classification based on MMR immunohistochemistry revealed MMRd in 70 patients (32.7%) and p53-abnormal expression in 10 patients (5.2%). Lymph node involvement was present in 23 patients (10.7%) (Table 1). During the study follow-up period (median 24.6 months, IQR: 24.7 months), a total of 15 death events were recorded.

**Table 1:** Clinicopathological characteristics of the study group

Variables			p-value
Age at diagnosis (years), mean±SD			60.61 ± 9.70
Body mass index (kg/m²), mean±SD			32.44 ± 6.19
Tumor size (mm), median [IQR]			12.0 [8.0–22.0]
Variables			n(%)
FIGO stage			
	Stage I		75(75.8)
	Stage II		13(13.1)
	Stage III		9(9.1)
	Stage IV		2(2.0)
Tumor grade			
	Grade 1 (G1)		94(46.1)
	Grade 2 (G2)		85(41.7)
	Grade 3 (G3)		25(12.3)
Histological subtype			
	Endometrioid		197(92.1)
	Serous		4(1.9)
	Clear cell		1(0.5)
	Carcinosarcoma		7(3.3)
	Other		5(2.3)
Lymphovascular space ınvasion (LVSI)			
	Absent		172(80.4)
	Present		42(19.6)
		Focal	26(12.1)
		Substantial	16(7.5)
Molecular classification			
	MMRd		70(32.7)
	p53abn		10(5.2)
Lymph node ınvolvement			
	Absent		191(89.3)
	Present		23(10.7)
Follow-up time (months), median [IQR]			24.6[24.7]
Death events			15(7.0)

SD - standard deviation; IQR, interquartile range; FIGO - International Federation of Gynecology and Obstetrics; LVSI - lymphovascular space invasion; MMRd - mismatch repair deficient; p53abn, p53-abnormal; NSMP - no specific molecular profile. Data are expressed as mean±SD or n(%). A p value of <0.05 indicates a significant difference

### Association of LVSI with Clinicopathological characteristics

Comparative analysis of clinicopathological features stratified by LVSI status is presented in table 2. Patients with LVSI-positive tumors (n = 42) demonstrated significantly more aggressive clinicopathological features compared to those without LVSI (n = 172). The presence of LVSI was significantly associated with higher tumor grade, with high-grade (G3) tumors observed in 28.6% of LVSI-positive cases versus only 7.6% of LVSI-negative cases (*p*<0.001). Non-endometrioid histology was more common in LVSI-positive tumors (21.4%) compared to LVSI-negative tumors (4.7%, *p*<0.001). Tumor size was significantly larger in the LVSI-positive group (median 34.0 mm, IQR: 20.0–50.0 mm) compared to the LVSI-negative group (median 10.0 mm, IQR: 7.0–16.0 mm; *p*<0.001). Lymph node involvement was more frequent in patients with LVSI-positive tumors (40.5%) compared to those with LVSI-negative tumors (2.9%, *p*<0.001). Early-stage disease (FIGO stage I–II) was significantly less common among LVSI-positive patients (21.4%, n=9/42) compared to those without LVSI (46.2%, n=79/172; *p*<0.001). The distribution of MMRd did not differ significantly between LVSI-positive and LVSI-negative groups (33.3% vs. 32.7%, respectively; *p*=0.924).

**Table 2:** Comparison of clinicopathological parameters and survival outcomes by LVSI status

Variables	LVSI-Negative (n=172)	LVSI-Positive (n=42)	p-value
Age at diagnosis (years), mean±SD	60.44 ± 9.91	61.33 ± 8.85	0.592
BMI (kg/m²), mean±SD	32.14 ± 5.93	33.64 ± 7.10	0.159
Tumor size (mm), median [IQR]	10.0 [7.0–16.0]	34.0 [20.0–50.0]	<0.001
Early-stage disease (FIGO I–II)	79(46.2)	9(21.4)	<0.001
High-grade tumors (G3)	13(7.6)	12(28.6)	<0.001
Non-endometrioid histology	8(4.7)	9(21.4)	<0.001
Lymph node involvement	5(2.9)	17(40.5)	<0.001
MMRd molecular subgroup	56(32.7)	14(33.3)	0.924

SD - Standard Deviation, BMI - Body Mass Index, FIGO - International Federation of Gynecology and Obstetrics, LVSI - Lymphovascular Space Invasion, MMRd - Mismatch Repair Deficient. Data are expressed as mean±SD or n(%). A p-value of <0.05 indicates a significant difference. Early-stage disease defined as FIGO stage I–II. High-grade tumors defined as Grade 3 (G3) histology

Multivariable Cox proportional hazards regression analysis was performed to assess the independent prognostic significance of LVSI in the overall cohort, adjusting for FIGO stage, tumor grade, histologic subtype, and MMR status (Table 3). In the adjusted model, LVSI demonstrated a trend toward increased risk of death (HR: 3.06, 95% CI: 0.54–17.44, *p*=0.208), although this association did not reach statistical significance. The wide confidence interval and lack of statistical significance likely reflect the limited number of death events (n=15) in this predominantly early-stage cohort, which substantially restricted statistical power to detect moderate effect sizes. Other variables in the multivariable model, including tumor grade, FIGO stage, MMRd status, and histologic subtype, similarly failed to demonstrate statistically significant independent prognostic effects, further underscoring the limitation imposed by small event numbers.

**Table 3:** Multivariable Cox proportional hazards regression analysis

Variables	Hazard ratio (HR)	95% Confidence ınterval	p-value
LVSI (positive vs. negative)	3.060	0.537–17.439	0.208
Tumor grade (G1 vs. G0)†	1.186	0.104–13.521	0.891
Tumor grade (G2 vs. G0)†	2.402	0.235–24.600	0.460
FIGO stage (advanced vs. early)‡	NS	NS	NS
MMRd (deficient vs. proficient)	1.227	0.321–4.692	0.765
Histology (non-endometrioid vs. endometrioid)	NS	NS	NS
Lymph node metastasis (present vs. absent)	2.520	0.359–17.679	0.352

† G0 represents reference category for tumor grade comparisons.

‡ NS: Not significant; specific values not shown for non-significant variables with extreme confidence intervals. SD - standard deviation; BMI - body mass index; IQR - interquartile range; FIGO - International Federation of Gynecology and Obstetrics; G3 - Grade 3; MMRd - mismatch repair deficient; LVSI - lymphovascular space invasion; HR - hazard ratio; NS - not significant

### Prognostic value of LVSI within the MMRd molecular subgroup

Detailed clinicopathological characteristics and prognostic analyses specific to the MMRd subgroup are presented in table 4.

**Table 4:** Clinicopathological characteristics and prognostic associations of LVSI within the MMRd molecular subgroup

Variables	LVSI-Negative (n =56)	LVSI-Positive (n =14)	p-value
Age at diagnosis (years)	62.61 ± 9.27	58.00 ± 6.46	0.084
BMI (kg/m²)	31.86 ± 5.40	31.88 ± 5.28	0.990
Tumor size (mm)	14.94 ± 14.29	72.77 ± 100.64	<0.001
Early-stage disease (FIGO I–II)	20 (35.7)	1 (7.1)	0.011
High-grade tumors (G3)	5 (8.9)	5 (35.7)	0.010
Non-endometrioid histology	2 (3.6)	1 (7.1)	0.555
Lymph node involvement	2 (3.6)	6 (42.9)	<0.001

SD - Standard Deviation; BMI - Body Mass Index; FIGO - International Federation of Gynecology and Obstetrics; LVSI - Lymphovascular Space Invasion; MMRd - Mismatch Repair Deficient. Data are expressed as mean±SD or n (%). A p value of <0.05 indicates a significant difference. Early-stage disease defined as FIGO stage I–II. High-grade tumors defined as Grade 3 (G3) histology

Among 70 patients with MMRd endometrial cancer, the mean age at diagnosis was 61.69 ± 8.93 years and the mean BMI was 31.86 ± 5.34 kg/m². The mean tumor size was 10.5 mm (IQR: 8.0–18.0 mm). The majority of MMRd cases were diagnosed at an early FIGO stage, with 17 patients (24.3%) classified as stage I, 4 patients (5.7%) as stage II, 3 patients (4.3%) as stage III, and 1 patient (1.4%) as stage IV. Endometrioid histology predominated in the MMRd subgroup (95.3%, n=66/70). Tumor grade distribution was as follows: Grade 1 (35.7%, n=25), Grade 2 (45.7%, n=32), and Grade 3 (14.3%, n=10). LVSI was identified in 14 of 70 MMRd patients (20.0%), with equal distribution of focal and substantial involvement (n=7 each, representing 10.0% of the MMRd cohort).

Within the MMRd subgroup, LVSI-positive tumors exhibited significantly more aggressive clinicopathological characteristics compared to LVSI-negative tumors. Tumor size was significantly larger in LVSI-positive cases (median 34.8 mm, IQR: 9.7–72.6 mm) compared to LVSI-negative cases (median 9.7 mm, IQR: 12.6 mm; *p*<0.001). High-grade (G3) histology was significantly more common in LVSI-positive MMRd tumors (35.7%) than in LVSI-negative MMRd tumors (8.9%, *p*=0.010). Early-stage disease (FIGO stage I–II) was less frequent in the LVSI-positive group (7.1%, n=1/14) compared to the LVSI-negative group (35.7%, n=20/56; *p*=0.011). Lymph node involvement was more prevalent in LVSI-positive MMRd cases (42.9%, n=6/14) compared to LVSI-negative MMRd cases (3.6%, n=2/56; *p*<0.001). No significant differences were observed between LVSI-positive and LVSI-negative MMRd patients with respect to age at diagnosis (*p*=0.084), BMI (*p*=0.990), or histologic subtype (*p*=0.555).

Multivariable Cox proportional hazards regression analysis was performed within the MMRd subgroup to assess the independent prognostic effect of LVSI on overall survival, adjusting for FIGO stage, tumor grade, and histologic subtype. In the adjusted model, LVSI did not demonstrate a statistically significant independent prognostic effect on overall survival (HR: 2.11, 95% CI: 0.11–39.71, *p*=0.617). Similarly, other clinicopathological variables including FIGO stage, tumor grade, and histologic subtype failed to reach statistical significance in the multivariable model.

Kaplan-Meier survival analysis stratified by LVSI status within the MMRd subgroup demonstrated no statistically significant difference in overall survival distributions between LVSI-positive and LVSI-negative patients (log-rank test: χ²=1.552, df=1, *p*=0.213). While the survival curves suggested a trend toward worse outcomes in the LVSI-positive group, this difference did not reach statistical significance.

## Discussion

This retrospective study provides novel insights into the clinicopathological associations and prognostic implications of LVSI within the MMRd molecular subtype of EC. Consistent with prior reports, LVSI was significantly associated established markers of biological aggressiveness, including larger tumor size, higher histologic grade, advanced FIGO stage, and lymph node metastasis.^(14,15)^ Importantly, these clinicopathological associations persisted within the MMRd molecular subgroup, reinforcing the role of LVSI as a marker of adverse pathological features even in the context of molecularly defined disease subtype.^(16)^

A key finding of this study is that the presence of LVSI did not correspond to a statistically significant difference in overall survival among MMRd patients. However, these observations must be interpreted with considerable caution as exploratory, hypothesis-generating findings rather than definitive conclusions. The limited number of LVSI-positive MMRd cases and the small number of death events within this subgroup substantially restricted statistical power to detect clinically meaningful survival differences. Our findings align with recent molecularly stratified studies that have begun to dissect the prognostic value of traditional clinicopathological factors within specific molecular subgroups. Siegenthaler et al.^(16)^ reported that while LVSI adversely affected recurrence-free survival in MMRd tumors, it did not significantly influence overall survival, a pattern consistent with our observations. This divergence between effects on recurrence and overall survival may reflect the inherent limitations of OS as an endpoint in early-stage, molecularly favorable disease, where deaths are infrequent and competing causes of mortality may obscure cancer-specific effects.

Contemporary data from The Cancer Genome Atlas (TCGA) follow-up analyses and preliminary reports from ongoing randomized trials such as PORTEC-4a further underscore the complexity of integrating molecular classification with traditional clinicopathological risk factors in endometrial cancer.^(17,18)^ These studies emphasize that molecular subtype may modify the prognostic impact of features such as LVSI, tumor grade, and depth of invasion, necessitating molecular subtype-specific risk stratification algorithms. The ongoing RAINBO clinical trial program^(17)^ specifically aims to refine adjuvant treatment recommendations based on integrated clinicopathological-molecular risk assessment, and will provide prospective data to guide clinical decision-making in molecularly defined subgroups.

This study possesses several methodological strengths that enhance the validity and reliability of the findings. First, all cases underwent systematic re-review of LVSI status by two gynecologic pathologists who were blinded to clinical outcomes, with independent classification of LVSI into focal and substantial categories according to contemporary standardized criteria.^(12)^ This approach minimizes inter-observer variability and assessment bias, which are recognized limitations in retrospective pathology studies. Second, molecular classification was performed using validated immunohistochemical protocols with appropriate internal controls, consistent with WHO 5th edition guidelines.^(13)^ Despite these strengths, this investigation is subject to several important limitations inherent to its retrospective, single-center design that must be carefully considered when interpreting the results. The limited number of LVSI-positive MMRd cases (n=14) and the small number of death events (n=15 in the overall cohort) substantially restricted statistical power to detect moderate effect sizes in survival analyses. The absence of POLE exonuclease domain sequencing in Turkey during the study period (2017–2020), and its continued unavailability in routine clinical practice at present, precluded complete four-group molecular classification of the cohort. As a result, we were unable to assign cases to the POLE-ultramutated molecular subgroup, which is associated with excellent prognosis despite often exhibiting high-grade histology.^(3)^ This limitation prevents direct comparison with studies employing complete molecular classification and may have resulted in misclassification of some POLE-mutated cases as NSMP or MMRd. The relatively short median follow-up time (24.6 months) limits the ability to assess long-term overall survival outcomes, particularly in early-stage, molecularly favorable disease where late recurrences may occur. Furthermore, recurrence-free survival (RFS) and progression-free survival (PFS) data were not systematically available for all patients due to incomplete documentation in the retrospective setting, particularly for patients who received follow-up care at external institutions. While OS was selected as the primary endpoint due to its reliability and lack of misclassification, RFS and PFS are generally more sensitive endpoints in early-stage endometrial cancer and would have provided earlier signals of treatment failure or biological aggressiveness. The inability to comprehensively analyze these time-to-event outcomes represents a significant limitation that reduces the clinical applicability of the findings. The ongoing molecularly stratified randomized trials, including PORTEC-4a^(18)^ and the RAINBO trial program,^(17)^ will provide prospective data to inform evidence-based, molecularly tailored treatment recommendations and should specifically examine the role of LVSI as a prognostic and predictive biomarker within molecular subgroups.

## Conclusion

This retrospective study demonstrates that lymphovascular space invasion is significantly associated with aggressive clinicopathological features in endometrial cancer, with these associations persisting within the MMRd molecular subgroup. However, LVSI did not independently influence overall survival in MMRd patients. Larger multicenter studies with complete molecular classification, standardized long-term follow-up are needed to validate these observations and to clarify the prognostic role of LVSI in molecularly defined endometrial cancer subgroups.

## Data Availability

The research data are described in the article presented.
